# Carboxymethylcellulose/Hydrotalcite Bionanocomposites as Paraben Sorbents

**DOI:** 10.1021/acs.langmuir.2c03265

**Published:** 2023-04-06

**Authors:** Daniel Cosano, Dolores Esquivel, Francisco J. Romero-Salguero, César Jiménez-Sanchidrián, José Rafael Ruiz

**Affiliations:** Departamento de Química Orgánica, Instituto Químico para la Energía y el Medioambiente (IQUEMA), Facultad de Ciencias, Universidad de Córdoba, Campus de Rabanales, Edificio Marie Curie, E-14071 Córdoba, Spain

## Abstract

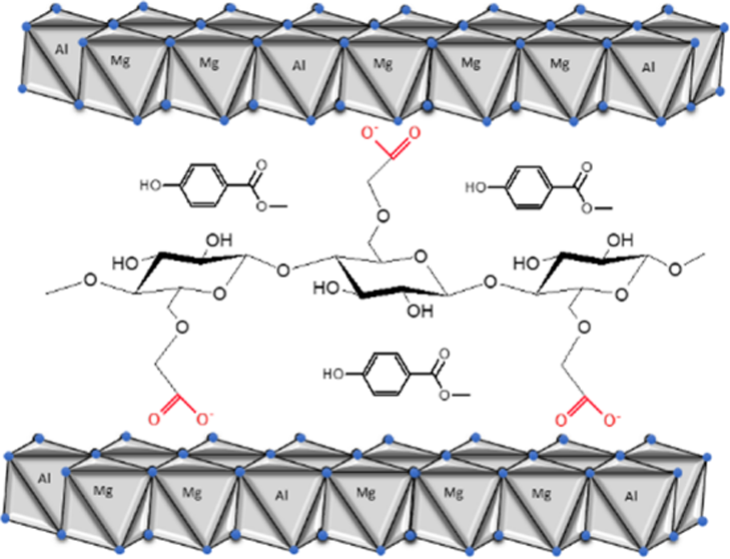

In this work, we synthesized several bionanocomposites of hydrotalcites containing carboxymethylcellulose as interlayer anion (HT-CMC) to be used as sorbents for parabens, a family of emergent pollutants (specifically, for 4-methyl*-*, 4-propyl*-* and 4-benzylparaben). Bionanocomposites were obtained by ultrasound-assisted coprecipitation and characterized by X-ray diffraction analysis, fourier transform infrared and raman spectroscopies, elemental and thermogravimetric analysis, scanning and transmission electron microscopies and X-ray fluorescence. All materials proved to be efficient sorbents for parabens through a process conforming to a pseudo second-order kinetics. The experimental adsorption data fitted the Freundlich model very closely and were also highly correlated with the Temkin model. The effects of pH, adsorbate concentration, amount of sorbent and temperature on the adsorption process was evaluated, obtaining the best results for methylparaben adsorption at pH 7, 25 mg of adsorbent and 348 K. The sorbent, HT-CMC-3, showed the highest adsorption capacity (>70%) for methylparaben. Furthermore, a reusability study showed that the bionanocomposite is reusable after its regeneration with methanol. The sorbent still retained its adsorption capacity for up to 5 times with a little loss of efficiency (<5%).

## Introduction

Until fairly recently, research into the environmental impact of chemicals focused on heavy metals, pesticides, and persistent organic compounds mainly. More recently, however, so-called “emergent pollutants” have aroused increasing interest among scientists on account of their potentially serious effects on human health and the environment. In fact, emergent pollutants are legally unregulated chemical substances with uncertain potentially adverse environmental effects.^1^ Although their presence in the environment is not new, it is only recently that awareness of their potential dangers has arisen. One salient property of these pollutants is that they need not be persistent to have deleterious effects on the environment. In fact, however rapidly they are removed, they continue to reach the environment virtually continuously. There are 12 categories of emergent pollutants. One encompasses endocrine-disrupting chemicals (EDCs), to which so-called “parabens” belong.^2^

Parabens are *p*-hydroxybenzoic acid esters typically used as additives and/or antimicrobial agents in cosmetic and pharmaceutical formulations and also in foodstuffs.^3^ The most widely used parabens are short-chain alkyl esters (largely methyl-, ethyl-, propyl-, and butylparabens). Recent studies have shown parabens to have deleterious effects on estrogens in males^4,5^ and to induce breast cancer in females.^6,7^ Removing parabens from wastewater has thus become essential to preserve not only aquatic life and the environment but also human health. A number of methods based on oxidation,^8^ photocatalysis,^9^ and decomposition reactions^10^ have been used to remove parabens from wastewater. One widely used method for this purpose involves adsorption onto silica,^11,12^ a filtering membrane^13^ or a clayey solid such as an anionic clay.^14−17^ Especially prominent in this respect are layered double hydroxides (LDHs), also known as “hydrotalcites” after their parent material: hydrotalcite [Mg_6_Al_2_(OH)_2_CO_3_·4H_2_O]. This mineral consists of stacked octahedral layers similar to those of brucite [Mg(OH)_2_], where substitution of a certain number of Mg^2+^ ions by Al^3+^ ions results in a charge deficiency that is offset by carbonate anions present in the interlayer region.^18^

Hydrotalcites containing a variety of di- and trivalent cations, and interlayer anions, can be easily synthesized.^19,20^ Their general formula is M(II)_1–*x*_M(III)_*x*_[(OH)_2_]^*x*+^[X_*n*/*x*_]^*n*−^·*m*H_2_O, where M(II) is a divalent cation, M(III) a trivalent cation, X the interlayer anion and *x* the metal ratio, M(III)/[M(II) + M(III)], which usually ranges from 0.20 to 0.40. Natural hydrotalcites typically have *x* = 0.33, which corresponds to an M(II)/M(III) ratio of 3; some, however, have a different ratio and are still stable.^21^ The anion can be of any type, whether organic and inorganic, or even a polysaccharide.^22,23^

Hydrotalcites are especially useful for adsorbing cadmium,^24−26^ chromium,^27^ and lead,^28−30^ among other species. Also, hydrotalcites functionalized with graphene oxide^31^ have been employed as sorbents of parabens. More recently, chitosan-Ni/Fe LDH composite was used as a solid-phase sorbent for the extraction of different parabens from standard and real samples.^32^ Through hydrophobic interactions between the hydrophobic part of chitosan and parabens, this type of material showed to be efficient over 30 reuse cycles. In this work, we assessed the potential of hydrotalcite-carboxymethylcellulose composites as paraben sorbents. In fact, hydrotalcites containing carboxymethylcellulose (CMC) have been successfully used as sorbents for various types of pollutants.^33−35^ Specifically, the hydrophobic interaction between CMC and parabens was previously shown to facilitate the adsorption of the latter into the composites. The impact of the interaction was explicitly observed on polymer–surfactant interactions by Schwuger and Lange,^36^ who found an especially strong hydrophobic interaction in carboxymethylcellulose (CMC). This was a result of CMC interacting with hydrotalcites—in fact, as shown in previous work, CMC by itself is a very poor sorbent for dissolved parabens.^37^

Therefore, the primary aim of this work was to obtain 2:1 Mg/Al hydrotalcites containing variable amounts of carboxymethylcellulose in the interlayer region by using a conventional coprecipitation method supplemented with ultrasound. The materials thus obtained were used as sorbents for parabens in aqueous solutions.

## Materials and Methods

### Reagents

The chemicals used, which included sodium carboxymethylcellulose (CMC), sodium hydroxide (NaOH, 99%), magnesium nitrate hexahydrate [Mg(NO_3_)_2_·6H_2_O], and aluminum nitrate nonahydrate [Al(NO_3_)_3_·9H_2_O], were all reagent-grade. Solutions of methyl 4-hydroxybenzoate, propyl 4-hydroxybenzoate, and benzyl 4-hydroxybenzoate (4-methyl-, 4-propyl- and 4-benzylparaben, respectively) were prepared by dissolving the required amounts of salt in de-ionized water.

### Synthesis of HT-CMC Bionanocomposites

Four different hydrotalcites (HTs) containing carboxymethylcellulose (CMC) anion in the interlayer region and one containing nitrate ion instead were prepared. Two of the CMC hydrotalcites were obtained by coprecipitation at low supersaturation and a constant pH, a procedure our group has frequently used to prepare HTs of diverse nature.^38−40^ In a typical run, a flask containing 4.5 g of sodium carboxymethylcellulose in 500 mL of water was supplied dropwise with 150 mL of an aqueous solution containing 0.05 mol of Mg(NO_3_)_2_·6H_2_O and 0.025 mol of Al(NO_3_)_3_·9H_2_O at a constant temperature of 60 °C over a period of 2 h under stirring to obtain a hydrotalcite with an Mg/Al ratio of 2 (i.e., *x* = 0.33). The pH was kept constant at 10 throughout by adding aqueous NaOH (1.5 M) as required. This procedure was used in duplicate, but the resulting materials were aged differently. Thus, one was heated at 80 °C for 24 h, whereas the other was placed under 0.2 Hz ultrasound for an identical length of time. After aging, the solids, which were named HT-CMC-1 and HT-CMC-2, respectively, were filtered off.

A third solid named HT-CMC-3 was obtained like HT-CMC-2 but using smaller amounts of Mg(NO_3_)_2_·6H_2_O (0.036 mol) and Al(NO_3_)_3_·9H_2_O (0.018 mol). Finally, the CMC-containing hydrotalcite HT-CMC-4 was obtained by coprecipitation under ultrasound energy. The coprecipitation process was similar to that for the previous solids; however, heating at 60 °C and stirring were replaced with the insertion of an ultrasonic probe operating at 0.2 Hz in the CMC solution. As a result, the temperature rose from 22 °C at the beginning to 30 °C after the metal salt was added. The resulting solid was filtered off but not aged.

A hydrotalcite containing nitrate as an interlayer anion named HT-NIT was also synthesized. The process was similar to that for HT-CMC-1 except that the solution supplied with the metal salts contained no CMC.

Once filtered, all hydrotalcites were washed with 2 L of bidistilled, de-ionized water.

### Adsorption of Parabens

Adsorption onto the hydrotalcite bionanocomposites was assessed by placing aqueous solutions of 4-methyl-, 4-propyl- and 4-benzylparaben in 100 mL flasks and adding 200 mg of one of the solids. All tests were performed at pH 7, which was that of the paraben solution, because other values led to worse results (see Figure S1). Also, all tests were performed under constant stirring at room temperature, and samples were periodically withdrawn for ultraviolet–visible (UV–vis) spectrophotometric analysis on a Suzi 455 instrument equipped with a tungsten lamp and a silicon photodiode detector. The three parabens showed the same absorbance maximum, so the wavelength was set at 255 nm (Figure S2). Once the optimum conditions for the process were established, adsorption tests were conducted with 4-methylparaben at variable concentrations and temperatures.

### Characterization of Solids

The five hydrotalcites studied were characterized by X-ray diffraction (XRD) and Raman spectroscopy, thermogravimetric analysis, and elemental and X-ray fluorescence (XRF) spectroscopy. XRD patterns were recorded over the 2θ range 2–70° by using Cu Kα radiation on a Siemens D-5000 spectrometer, and Raman spectra were acquired over the wavenumber range 140–1700 cm^–1^ by using green laser light (532 nm) on a Raman Renishaw spectrophotometer equipped with an InVia microscope. All spectral processing (baseline correction, smoothing) was done with the software Wire v.3.4 from Renishaw. Fourier transform infrared (FT-IR) spectroscopy was used from 4000 to 250 cm^–1^ on a FT-IR Nicolet Magna IR 500 instrument. Thermogravimetric analyses (TGA) were done by using a Setaram SetSys 12 analyzer. Measurements were made on 20 mg samples that were placed in an alumina crucible and heated from 30 to 800 °C at 10 °C min^–1^ under an air stream flowing at 50 mL min^–1^. Scanning electron micrographs and energy-dispersive spectra were obtained with a JEOL JSM 7800F microscope at a voltage of 15 kV and a distance of 10 mm. High-resolution transmission electron micrographs were obtained with a JEOL JEM 1400 microscope.

### Recyclability

The recyclability of the carboxymethylcellulose-hydrotalcite bionanocomposites was assessed in the sorbent with the highest adsorption capacity. Thus, an aqueous solution of 4-methylparaben (25 mg/L) was placed in a 100 mL flask and supplied with 200 mg of HT-CMC-3, after which the solid was filtered off and washed with de-ionized water and methanol. Then, the sorbent was regenerated by stirring in methanol (50 mL) for 3 h. This procedure was repeated after each use. As before, all tests were performed under constant stirring at room temperature in triplicate.

## Results and Discussion

### Characterization of the Sorbents

Table 1 shows the Mg/Al ratio of each hydrotalcite bionanocomposite as estimated from the XRF results and also its percent C and N contents as determined by elemental analysis. As can be seen, the metal ratio of each hydrotalcite was similar to the theoretical value irrespective of the way it was prepared. As can be inferred from the elemental analysis results, the amount of carbon present, which was equivalent to that of CMC, differed with the synthetic method, the hydrotalcites aged by sonication (viz., HT-CMC-2 and HT-CMC-3) being those containing the greatest amounts. Obviously, HT-CMC-3 contained a higher proportion of C than did HT-CMC-2 because the former was obtained from a greater amount of CMC. On the other hand, the hydrotalcite directly obtained under sonication was that containing the lowest proportion of C, possibly by effect of the ultrasound treatment partly decomposing CMC.^41^

**Table 1:** Composition of the Hydrotalcites

hydrotalcite	Mg/Al ratio^a^	%C^b^	%N^b^
HT-NIT	1.98		5.2
HT-CMC-1	2.03	18.9	0.8
HT-CMC-2	1.91	21.7	1.7
HT-CMC-3	1.93	25.5	1.4
HT-CMC-4	2.06	15.2	1.7

a Determined by XRF (molar ratio).

b Determined by CNS analysis (%w/w).

**Figure 1:**
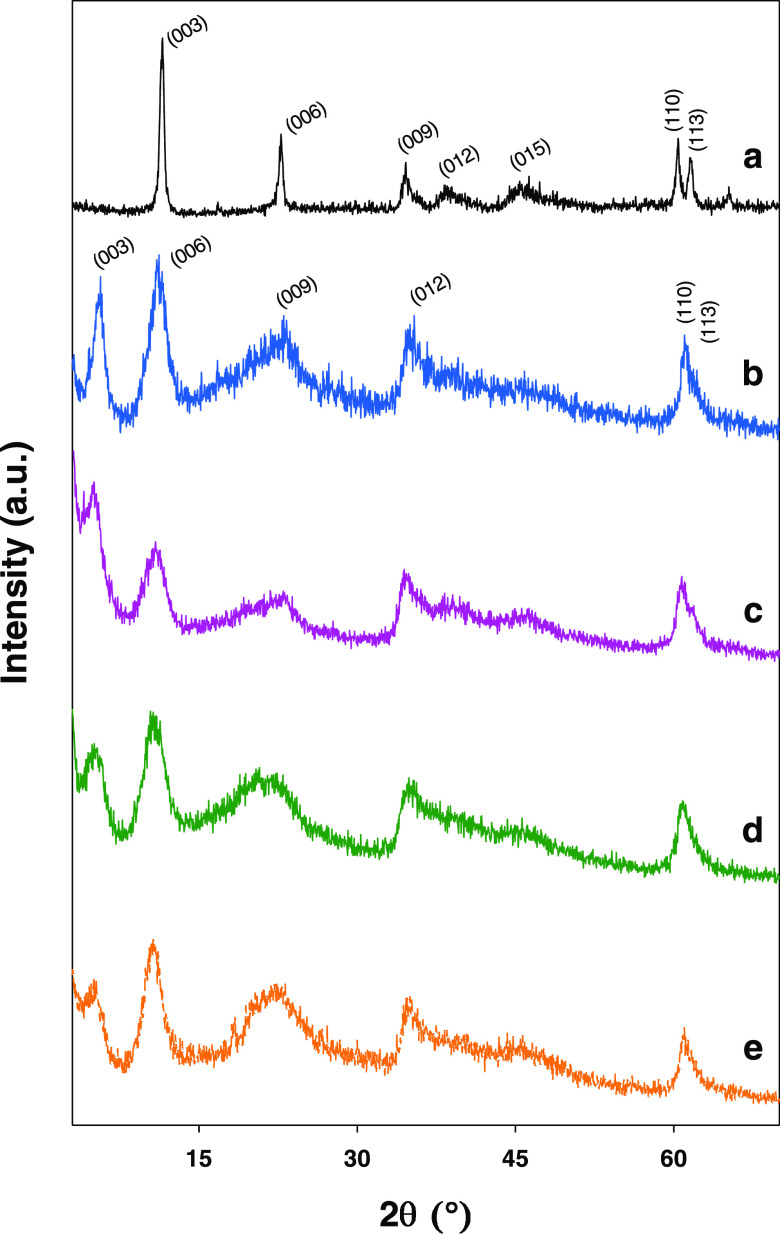
XRD patterns for the hydrotalcites. (a) HT-NIT, (b) HT-CMC-1, (c) HT-CMC-2, (d) HT-CMC-3 and (e) HT-CMC-4.

Figure 1 shows the XRD patterns for the hydrotalcites. The results for HT-NIT (Figure 1a) are consistent with a hydrotalcite containing interlayer nitrate anions to counter the charge deficiency. In fact, the (003), (006), (009), (012), (015), (110), and (113) lines are suggestive of a hydrotalcite phase^42^ with a hexagonal cell of rhombohedral symmetry (a 3R polytype) belonging to the *R*3̅*m* space group.

Also, the calculated lattice parameters (*c* and *a* in Table 2) are consistent with values previously determined by our group in similar solids.^43^ Parameter *c*, which is equivalent to three times the interlayer distance, was calculated as 3[*d*_(003)_ + 2*d*_(006)_]/2, and *a*, which is a measure of the distance between two neighboring cations in hydrotalcite octahedral layers, as 2*d*_(110)_.

**Table 2:** Lattice Parameters for the Hydrotalcites, All in Nanometers

hydrotalcite	*d*_(003)_	*d*_(006)_	*c*	*d*_(110)_	*a* nm
HT-NIT	0.867	0.441	2.632	0.153	0.306
HT-CMC-1	1.649	0.804	4.886	0.152	0.304
HT-CMC-2	1.797	0.814	5.138	0.153	0.306
HT-CMC-3	1.735	0.828	5.086	0.153	0.306
HT-CMC-4	1.748	0.826	5.100	0.152	0.304

As can be seen from Figure 1b–e, the presence of intercalated CMC had a considerable impact on the XRD spectra. Thus, it shifted baseline diffractions to lower 2θ values reflecting an increased interlayer distance. The increased width of the (003) and (110) lines for the hydrotalcite bionanocomposites relative to their nitrate counterpart suggests that the crystal dimensions in the *c* and *a* directions were reduced by the presence of the polymer in the interlayer region.^44^ Also, the (003) baseline spacing for the bionanocomposites ranged from 1.6 to 1.8 nm, and the (006) spacing from 0.80 to 0.83 nm; therefore, parameter *c* ranged from 4.8 to 5.2 nm and was thus similar to previously reported values for CMC-intercalated hydrotalcites.^33,45^ Judging by these results, the proposed synthetic method ensures efficient intercalation of CMC anion between hydrotalcite layers. Also, the presence of a broad halo at ca. 20° is suggestive of a poorly crystalline phase (possibly residual non-intercalated polymer^46^). Long alkyl chains may be partly intercalated and partly occupy the edge or outer surface of hydrotalcite particles.^47^

**Figure 2:**
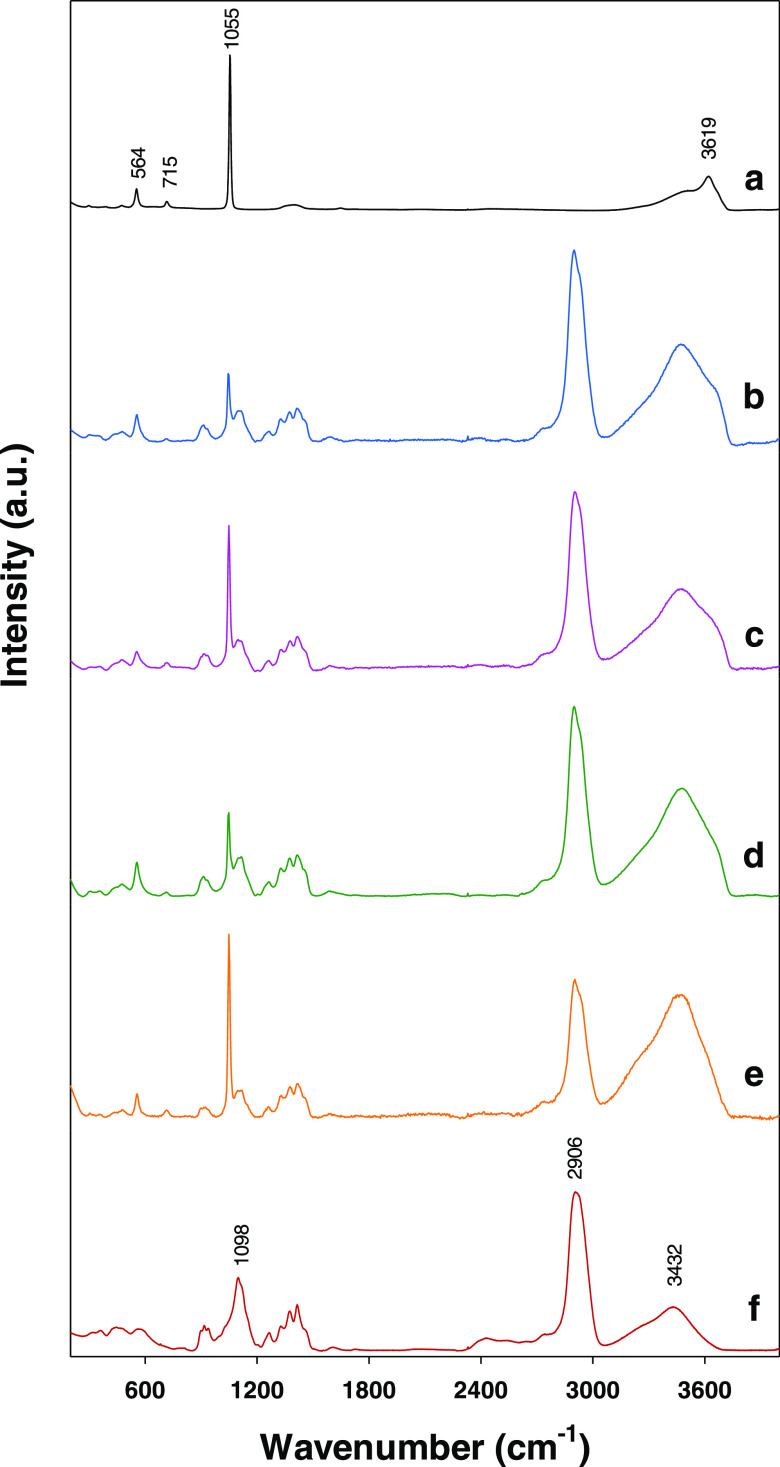
Raman spectra for the hydrotalcites: (a) HT-NIT, (b) HT-CMC-1, (c) HT-CMC-2, (d) HT-CMC-3, (e) HT-CMC-4. (f) Spectrum for sodium carboxymethylcellulose.

Based on the elemental analysis results, all CMC hydrotalcites contained a small amount of nitrogen, possibly as a result of nitrate in the metal salts reaching their interlayer region. This point was clarified by using Raman spectroscopy, which had previously allowed our group to characterize the interlayer anion^48,49^ and the nature of the hydroxyl groups^50,51^ in various hydrotalcites. Figure 2 shows the Raman spectra obtained here. The bands for hydrotalcite were identified by examining the spectrum for HT-NIT. The Raman spectrum for a hydrotalcite typically spans three different wavenumber regions, namely: 3100–3700, 1000–1700, and 200–800 cm^–1^, which is where O–H bond stretching vibrations, the main bands for intercalated anions and M–O stretching vibrations (with M = Al or Mg), respectively, appear. A fourth region (2750–3100 cm^–1^, which typically contains C–H vibration bands) is also examined when the intercalated anion is organic. The Raman spectral profile for HT-NIT in the 3100–3700 cm^–1^ region was similar to others observed in previous work^50,51^ and contained three bands—results of the deconvolutive analysis not shown—at ca. 3105, 3312, and 3619 cm^–1^ that can be assigned to stretching vibrations in O–H bonds forming hydrogen bonds with interlayer nitrate anions, O–H bonds in water molecules also present in the interlayer region and O–H bonds in octahedral layers, respectively.^52^ The 1000–1700 cm^–1^ spectral region exhibited a very strong band at 1055 cm^–1^ due to stretching of N–O bonds in nitrate groups.^51^ Finally, the 200–800 cm^–1^ region contained two weak bands at 564 and 715 cm^–1^ that were assigned to asymmetric stretching of Al–OH bonds^52^ and Mg–O vibrations,^53^ respectively.

**Figure 3:**
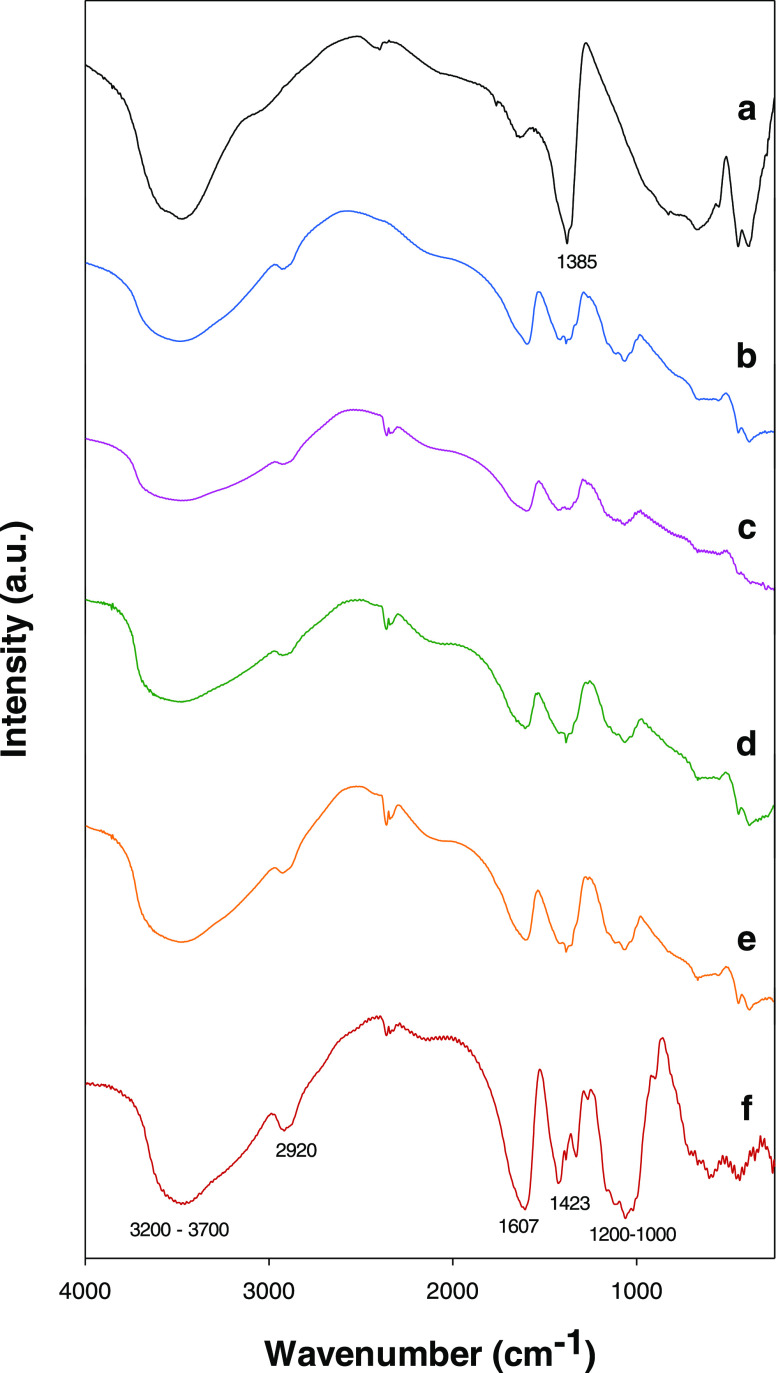
FT-IR spectra for the hydrotalcites: (a) HT-NIT, (b) HT-CMC-1, (c) HT-CMC-2, (d) HT-CMC-3, (e) HT-CMC-4. (f) Spectrum for sodium carboxymethylcellulose.

Figure 2b–e shows the spectra for the CMC-containing hydrotalcites and Figure 2f shows that for sodium carboxymethylcellulose. As can be seen, the spectra for the HT-CMC solids were virtually identical and differed only in the strength of some bands. One salient feature was the increased strength of the broadband at 3100–3700 cm^–1^ resulting from the presence of hydroxyl groups in the intercalated polymer. A comparison of the spectrum for sodium carboxymethylcellulose and those for the CMC hydrotalcites revealed the presence of the bands for CMC anion and a shoulder at ca. 3620 cm^–1^ due to OH groups in metal layers. Immediately below 3000 cm^–1^ was a very strong band due to stretching of C–H bonds of methylene groups in CMC molecules. Also, the region from 1000 to 1400 cm^–1^ contained a well-defined band for nitrate that confirmed its presence in addition to others that were ascribed to C–C and C–O stretching vibrations and C–H bending vibrations in CMC molecules. Finally, the spectra for the HT-CMC solids also contained bands for stretching vibrations of Al–OH and Mg–O bonds in metal layers. These results were further corroborated by FT-IR spectroscopy (Figure 3). The FT-IR spectrum of CMC exhibited a broad band in the range from 3200–3700 cm^–1^, attributed to the stretching of −OH groups and hydrogen bonds. The C–H stretching vibration of the ring of cellulose was present at 2920 cm^–1^. Additional peaks at 1423 and 1607 cm^–1^ corresponded to the vibrations of the carboxylate groups. Also, the region from 1000 to 1200 cm^–1^ is assigned with the −C–O– stretching on the polysaccharide skeleton (Figure 3f).^54^ The FT-IR spectrum of HT-Nit showed three main wavelength ranges, corresponding to hydroxyl vibrations (3000–4000 cm^–1^), interlayer anion vibrations (1200–1800 cm^–1^), and lattice skeleton vibrations (below 1200 cm^–1^) (see Figure 3a). The intense peak centered at 1385 cm^–1^ corresponded to the N–O stretching vibration band for nitrate ion.^55^ HT-CMC bionanocomposites (Figure 3b–e) showed those vibration bands associated with the CMC.^56^ Compared to the FT-IR spectrum of HT-Nit, all bionanocomposites showed additional peaks at 2920 and1607 cm^–1^ attributed to the C–H and −C–O– stretching of the intercalated CMC, respectively.

**Figure 4:**
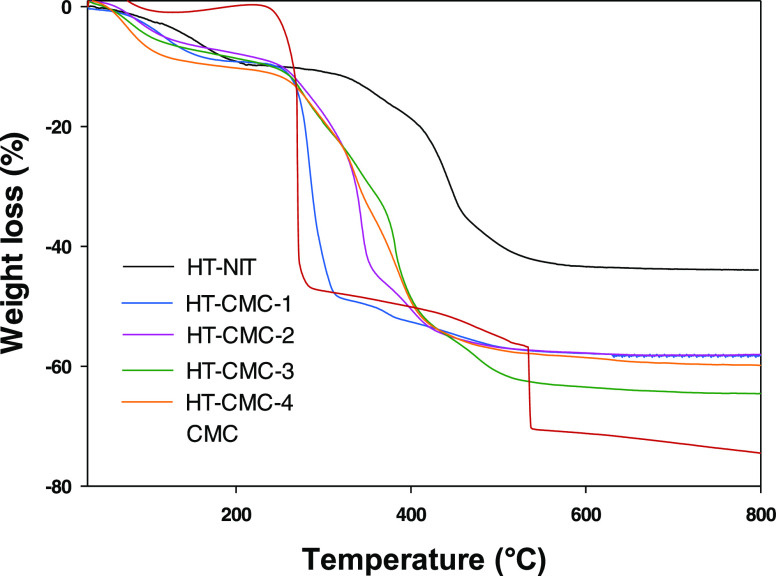
Thermogravimetric curves for the hydrotalcites and sodium carboxymethylcellulose.

The hydrotalcites were additionally characterized by thermogravimetric analysis (TGA). As can be seen from Figure 4, sodium carboxymethylcellulose exhibited two distinct thermal decomposition steps. Thus, up to 200 °C, CMC underwent dehydration by losing water molecules adsorbed onto hydrophilic chains in the polymer. From 250 to 500 °C, the polymer decomposed into carbon dioxide and water, which formed sodium carbonate, the carbonate in turn decomposed above 800 °C^57^ and gave a signal not shown in the figure.

On the other hand, the TGA curve for HT-NIT exhibited three distinct steps. The first (25–220 °C) was due to the loss of physisorbed water and water molecules in the interlayer region; the second (220–550 °C) to the structural decomposition of hydrotalcite into a magnesium–aluminum mixed oxide; and the third (above 550 °C) to a phase change from the previous oxide to a spinel (MgAl_2_O_4_) that resulted in a very small weight change.^38^ Intercalating CMC anion into hydrotalcite altered the TGA curves by effect of ionic and molecular interactions (see Figure 3) as previously found with other organic intercalates.^43^ As can be seen from Table 3, the HT–CMC hydrotalcites underwent a weight loss roughly 15% greater than that in HT-NIT—by exception HT-CMC-3 exhibited a greater loss (21%) as a result of its containing excess CMC.

**Table 3:** Percent Weight Loss in Each Step of the Thermogravimetric Curves

hydrotalcite	30–220 °C	220–550 °C	550–800 °C	30–800 °C
HT-NIT	8	32	4	44
HT-CMC-1	9	47	2	58
HT-CMC-2	9	47	2	58
HT-CMC-3	9	53	3	65
HT-CMC-4	10	46	3	59
CMC	0	70	5	75

Finally, the scanning electron microscopy (SEM) (Figure S3) and transmission electron microscopy (TEM) results (Figure S4) were essentially identical among samples, all of which had a platelet-like appearance.

### Kinetic Determinations

Once characterized, the hydrotalcites were assessed as paraben sorbents in kinetic terms. Tests were conducted by using volumes of 50 mL of paraben solutions containing 25 mg/L methylparaben, 5 mg/L propylparaben, or 1 mg/L benzylparaben, which coincided with their highest solubility in water at room temperature. The amount of paraben adsorbed by each hydrotalcite was calculated from the following equation
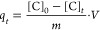
where *q*_*t*_ (mg paraben/g hydrotalcite) is the adsorption capacity of the hydrotalcite at time *t*, *V* (mL) the solution volume, *m* (g) the amount of hydrotalcite, *C*_0_ (ppm) the initial concentration of paraben, and *C*_*t*_ (ppm) that at time *t*.

**Figure 5:**
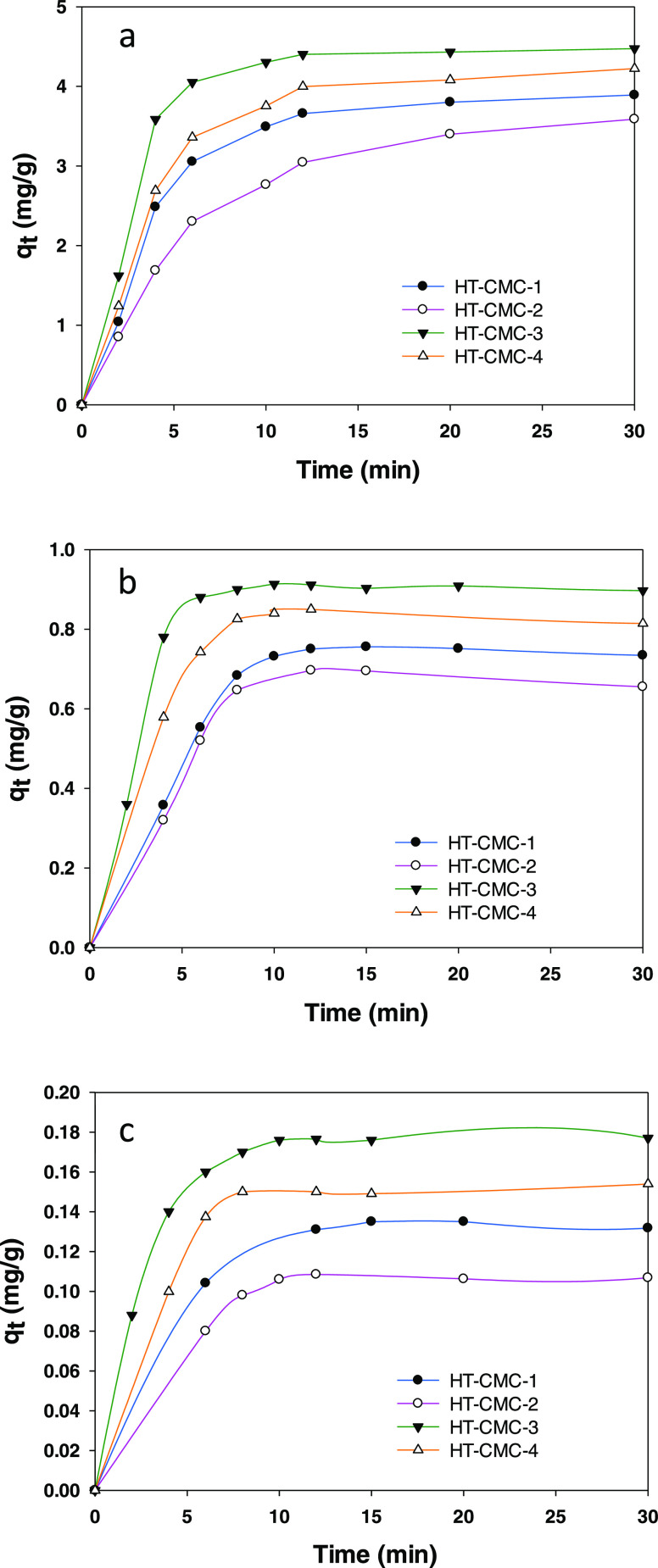
Adsorption of 4-methylparaben (a), 4-propylparaben (b), and 4-benzylparaben (c) onto various hydrotalcites bionanocomposites.

Figure 5 shows the kinetic curves for the parabens. Adsorption equilibrium was reached after 20 min. As can be seen, the curves for the HT-CMC hydrotalcites had a very steep slope at short times owing to the large number of free adsorption sites they contained; also, the paraben adsorption rate gradually decreased as such sites were occupied until equilibrium was reached.

The high initial rates of adsorption were a result of strong interactions between paraben molecules and CMC present on the sorbent surface; also, their subsequent decrease must have resulted from the parabens diffusing into the interlayer region of the sorbents. Therefore, the parabens were initially adsorbed at a high rate onto the outer surface of each solid and, once the surface saturated, paraben molecules migrated into hydrotalcite pores^58^ to be adsorbed at a much lower rate in the interlayer region. As can be seen from Figure 5, the slight crystallographic changes in the sorbents resulted in small differences among materials, the most efficient paraben sorbent being that containing the largest amount of CMC. These results are quite promising as regards using hydrotalcite-based composites as paraben sorbents. The absence of ionic adsorption was checked by using a nitrate-containing hydrotalcite. No paraben adsorption was observed with it. Therefore, the adsorbate was adsorbed onto the composite through hydrophobic interaction of the former with CMC in the latter. 4-Methylparaben, which was the most soluble paraben, was then used in various kinetic determinations.

### Kinetic Models

**Figure 6:**
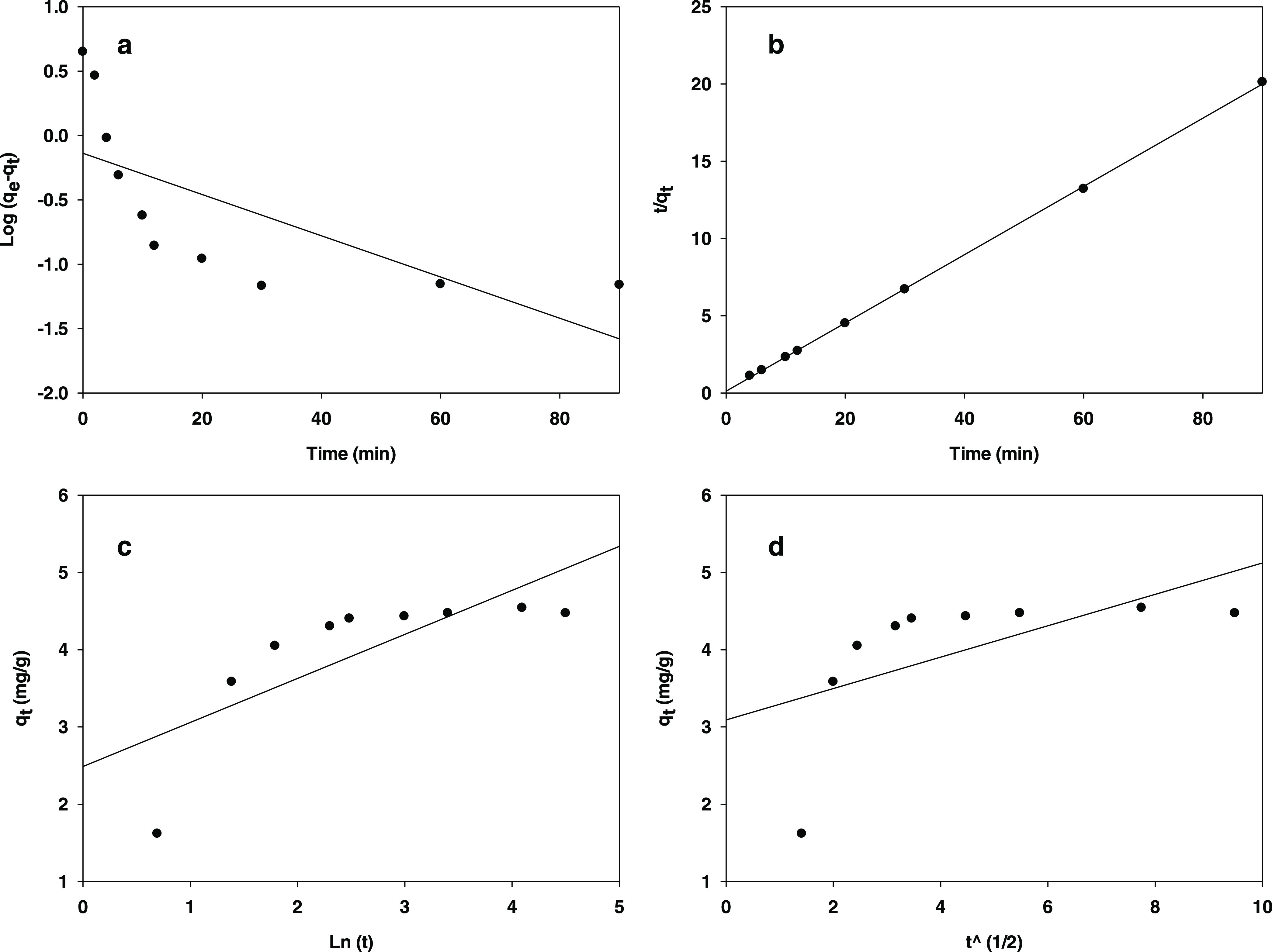
Kinetics of the adsorption process. (a) Pseudo first-order model. (b) Pseudo second-order model, (c) Elovich model, and (d) intraparticle diffusion model.

Adsorption of parabens onto the hydrotalcites was examined in the light of various kinetic models (Figure 6). The pseudo first-order model fitted eq 1, which corresponds to preferential adsorption in monolayer form
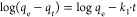
1*q*_e_ and *q*_*t*_ being the amount of paraben adsorbed per gram of hydrotalcite at equilibrium and time *t*, respectively, and *k*_1_ the pseudo first-order kinetic constant for the process (see Figure 6a).

The pseudo first-order model, which is defined by eq 2, assumes the adsorbate to be chemisorbed onto the sorbent
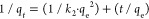
2Where, *q*_e_ and *q_t_* have the same meaning as in eq 1, and *k*_2_ is the pseudo sec ond-order rate constant for the process. As can be seen from Table 4, the results fitted this model quite closely, which suggests that the parabens were chemisorbed onto the hydrocarbon backbone of carboxymethylcellulose.

**Table 4:** Kinetic Adsorption Parameters for Removal of Methylparaben with HT-CMC-3

Pseudo first order			Pseudo second order		
*q*_e_ = 0.501	*K*_1_ = 0.0132	*R*^2^ = 0.4671	*q*_e_ = 4.519	*K*_1_ = 213.81	*R*^2^ = 0.9998
Elovich			intra particular diffusion		
*h*_b_ = 78.637	*B* = 1.754	*R*^2^ = 0.5787	*K*_intra_ = 0.2031	*c* = 3.0911	*R*^2^ = 0.4671

The Elovich model assumes a second-order adsorption kinetics involving chemisorption onto heterogeneous solid surfaces. The mathematical equation for the model is
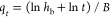
3where *h*_b_ and *B* are two constants. Based on the curve of Figure 6c, paraben molecules were initially adsorbed very rapidly and to a large extent and then diffused into the interlayer region of the hydrotalcites in a second step.

As confirmed by the intraparticle diffusion model, the parabens were chemisorbed onto the hydrotalcites. With intraparticle diffusion, the solute is carried through the pore network by diffusion. Intraparticle diffusion is defined by eq 4, where specific adsorption of the solute is proportional to the square root of time
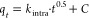
4*C* being the portion of solute adsorbed at the beginning of the process and *k*_intra_ the intraparticular diffusion rate constant. As can be inferred from the curve of Figure 6d, the adsorption process involved two steps, namely: saturation of the sorbent surface with the adsorbate and diffusion into sorbent cavities. The high slope of the initial portion suggests that the rate-determining step was diffusion of ionic species into the cavities of the material.^59^

### Influence of the Adsorbate Concentration

**Figure 7:**
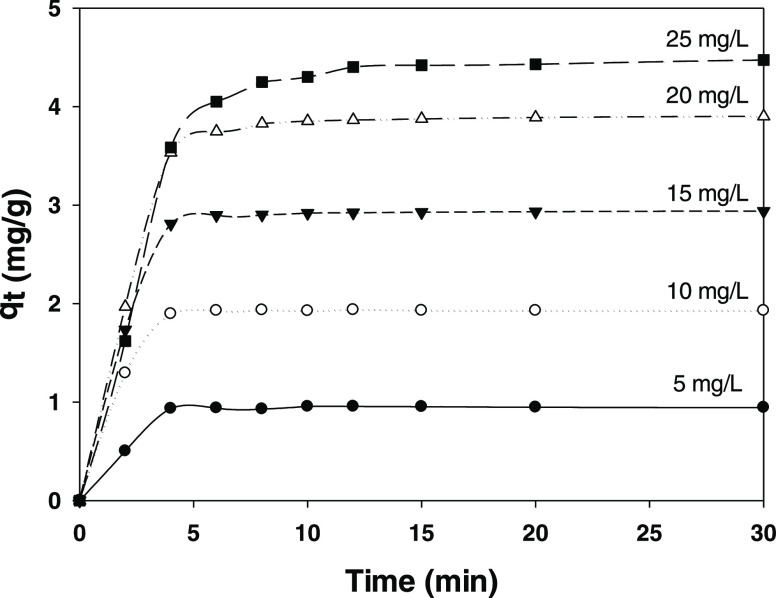
Influence of the initial concentration of 4-methylparaben on its adsorption onto HT-CMC-3. Experimental conditions: 50 mL of adsorbate solution, 200 mg of sorbent, and 22 °C.

Figure 7 shows the influence of the initial concentration of adsorbate on the adsorption of methylparaben onto HT-CMC-3, which had previously proved the most efficient sorbent. Tests were performed with initial concentrations of 5, 10, 15, 20, and 25 mg/mL on the constancy of all other conditions. As can be seen, the mass transfer rate increased with increasing paraben concentration and led to an increasing rate of adsorption onto the hydrotalcite surface^58^—and hence to an increasing amount of paraben being adsorbed. The fast initial adsorption observed can be ascribed to contact of paraben molecules with free surface sites in CMC; on the other hand, the subsequently low rate was a result of paraben molecules being adsorbed into sorbent pores. Based on the results, HT-CMC-3 is a good paraben sorbent candidate.

### Influence of the Amount of Sorbent

**Figure 8:**
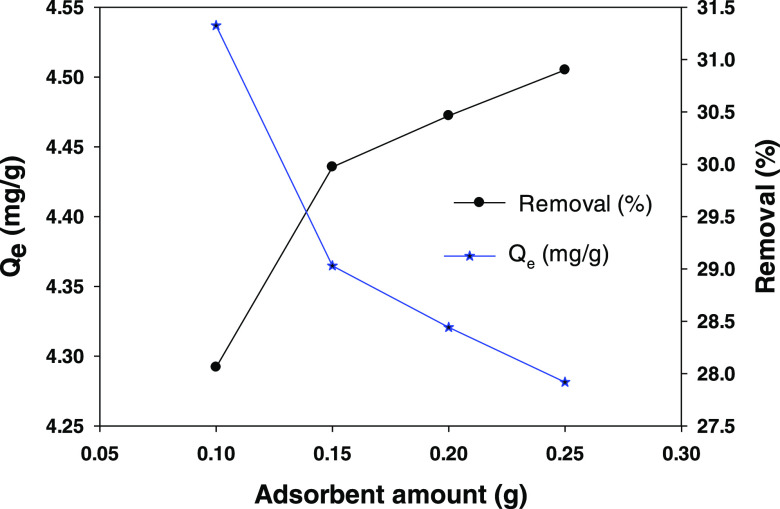
Influence of the amount of HT-CMC-3 on the adsorption of 4-methylparaben.

The influence of the amount of sorbent was examined by using variable amounts of HT-CMC-3 (0.10, 0.15, 0.20, and 0.25 g) and 50 mL of a 25 mg/L methylparaben solution at 22 °C. Figure 8 shows the influence of the amount of sorbent on the adsorption capacity (*Q*_e_) and removal efficiency (%*R*). Increasing the amount of sorbent used increased the number of sites available for the adsorption of parabens—and hence their removal efficiency. As can be seen from Figure 8, the increase in *Q*_e_ was not linear.

### Influence of Temperature

The influence of this operational variable on methylparaben adsorption onto HT-CMC-3 was examined at 295, 308, 323, and 348 K. Based on the results (not shown), the paraben adsorption rate increased markedly with increasing temperature, which testifies to the endothermic nature of the process, the activation energy for which was calculated from the Arrhenius equation in logarithmic form
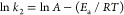
5

**Figure 9:**
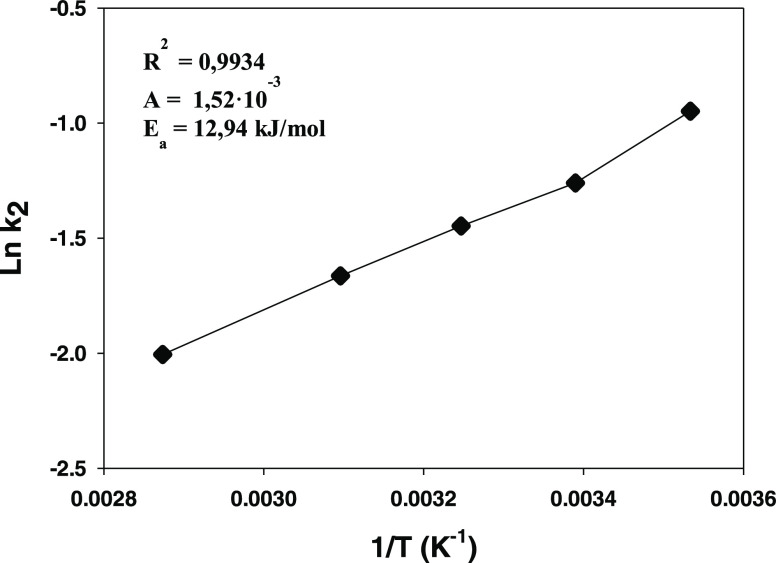
Variation of ln *k*_2_ with the reciprocal temperature.

In fact, a plot of ln *k*_2_ against the reciprocal temperature (Figure 9) was a straight line with a very high correlation coefficient from which an activation energy of 12.94 kJ/mol was calculated.

The results were used to calculate the thermodynamic quantities Δ*H*°, Δ*S*°, and Δ*G*° for the sorption system, using the linear form of the van’t Hoff equation^60^
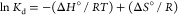
6
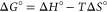
7Where Δ*H*°, Δ*S*° and Δ*G*° are the enthalpy, entropy, and free energy of adsorption, respectively; *T* is the absolute temperature, in kelvin; and *R* is the ideal gas constant (1.987 kcal mol^–1^). The Δ*H*° value thus obtained, −2615.97 kcal mol^–1^, indicates that the sorption process is exothermic. Also, the resulting entropy change, Δ*S*° = −7.948 kcal mol^–1^ K^–1^, is suggestive of an orderly system and hence of easy adsorption. Finally, the free energy change, Δ*G*° = −247.38 kcal mol^–1^, is typical of a spontaneous adsorption process.

### Adsorption Isotherms

**Figure 10:**
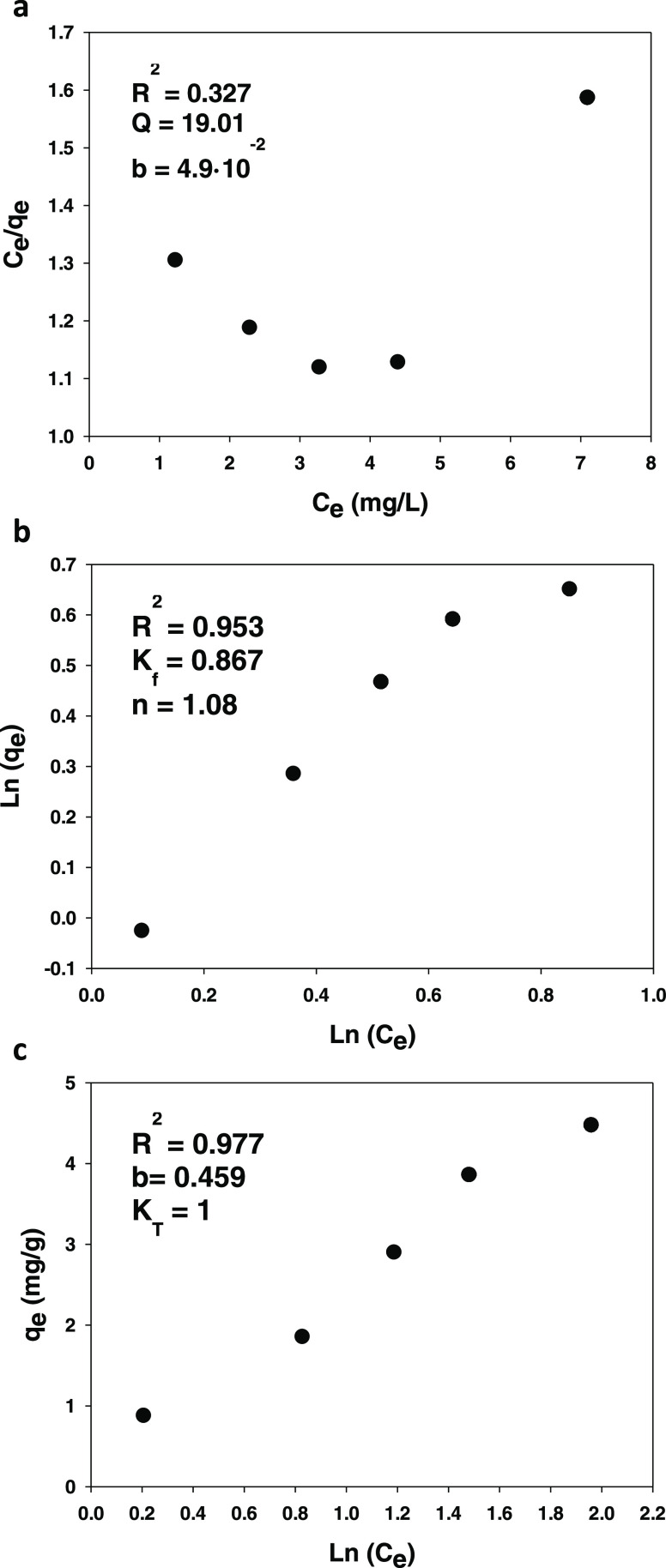
Langmuir (a), Freundlich (b), and Temkin plot (c) for 4-methylparaben adsorption.

The results obtained at 295 K in the previous tests were used to construct an adsorption isotherm for the process. The experimental data were fitted by using the three most common models for this purpose, which are based on the Langmuir, Freundlich, and Temkin isotherms (see Figure 10).

The Langmuir isotherm is defined by
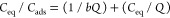
8where *C*_eq_ (mg/L) is the equilibrium adsorption concentration of methylparaben, *Q* (mg/g) the amount adsorbed in monolayer form per unit mass of sorbent, *C*_ads_ (mg/g) the *Q* value at equilibrium, and b a constant dependent on the affinity of binding sites. This model assumes homogeneous adsorption in monolayer form.

The Freundlich model is given by
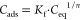
9

or, in logarithmic form
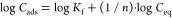
10where *K*_f_ is the adsorption capacity and *n* is the intensity of a given sorbent. The Freundlich model assumes heterogeneous adsorption in multiple layers.

Finally, the Temkin isotherm is defined by
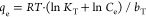
11where *K*_T_ and *b*_T_ are Temkin’s equilibrium binding constant (L/g) and adsorption heat constant, respectively. This model assumes a uniform distribution of binding energies and introduces constants dependent on the initial heat of adsorption, which is assumed to decrease linearly with increasing coverage. This model typically holds at medium concentrations of the adsorbate. Also, because the heat of adsorption of molecules in monolayer form is temperature-dependent, the model assumes that it decreases linearly rather than logarithmically with increasing coverage.^61^

Figure 10 shows the Langmuir, Freundlich, and Temkin isotherms for paraben adsorption onto the hydrotalcites. As can be seen, the results fitted the Freundlich model more closely than they fitted the Langmuir model. The fact that the correlation coefficient, *R*^2^, for the Freundlich model was greater indicates that paraben molecules were heterogeneously adsorbed in multilayer form onto the hydrotalcite surface. Also, the fact that *n* ranged from 1 to 10 further supports that adsorption conformed to the Freundlich model.^62^ Finally, the additional fact that the Temkin model also exhibited a high correlation coefficient is suggestive of sorbent–adsorbate interaction provided the heat of adsorption does not remain constant.^61^

### Adsorption Mechanism

**Scheme 1:**
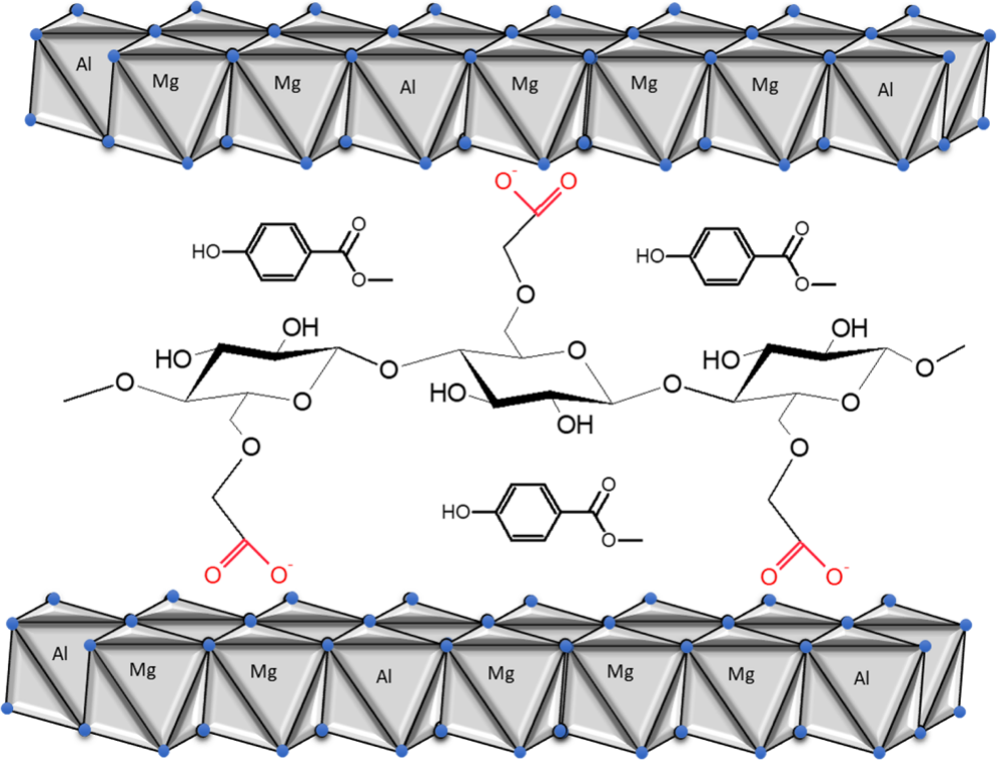
Hydrophobic Interaction between Methylparaben and Interlaced Cellulose in Hydrotalcite

The adsorption mechanism of hydrophobic organic compounds dissolved in water is not clearly described in the literature. It has been shown that the adsorption effect on parabens is correlated with the ratio of apolar and polar surface areas.^63^ This shows that those longer chain adsorbates, whose hydrophobicity increases, were positively correlated to the pollutant removal efficiency.^64^ Therefore, adsorption mechanism of parabens in the carboxymethylcellulose/hydrotalcite bionanocomposites is based on a hydrophobic interaction effect between the aromatic ring of parabens and cellulose chains (Scheme 1). This mechanism is in agreement with the mechanism generally accepted by other authors.^63,65^

### Recyclability

Recyclability was assessed by reusing the solid with the highest adsorption capacity (HT-CMC-3) to adsorb methylparaben. Figure S5 shows the outcome of 30 min adsorption cycles. As can be seen, the adsorption capacity of HT-CMC-3 remained similar to that of the fresh adsorbent after 5 cycles—it decreased by only 5% in each cycle. The solid was washed with methanol between cycles to desorb paraben^66^ because the hydrophobic interaction of the aromatic ring of paraben and cellulose chains in the material is known to produce weak bonds that are easily broken by the alcohol.^65,67^

## Conclusions

In this work, we synthesized various Mg/Al organo-hydrotalcites containing carboxymethylcellulose anion in the interlayer region. The solids were obtained by following a procedure based on conventional coprecipitation albeit with the aid of ultrasound energy in some cases. All resulting solids were layered materials with an Mg/Al metal ratio close to 2, which is the theoretical value. XRD patterns revealed the formation of organo-hydrotalcite phases; also, FT-IR and Raman spectra confirmed the presence of the polymer anion in the interlayer region in all composites.

The HT-CMC solids were assessed as sorbents for parabens in aqueous solutions. Overall, the bionanocomposites exhibited a high adsorption capacity for methyl-, propyl-, and benzylparaben. The modification of the method of synthesis and/or aging of the bionanocomposite does not directly affect the adsorption of the paraben, but this adsorption is directly related to the amount of intercalated CMC. Adsorption of the parabens was a pseudo second-order process where they were initially adsorbed at a very high rate onto CMC molecules anchored to the surface of outer brucite layers in the hydrotalcites. After such layers saturated with carboxymethylcellulose (CMC), adsorbate molecules diffused into interlayer CMC at a much lower rate. As revealed by different tests, the parabens were adsorbed by chemisorption.

The paraben adsorption efficiency of the hydrotalcite bionanocomposites was found to depend on the initial paraben concentration and amount of sorbent used. We also demonstrated that the optimum working pH was 7. By studying the influence of temperature, we verified that the adsorption process is spontaneous. Also, the process fitted the Freundlich isotherm model. The most efficient bionanocomposite (HT-CMC-3) was successfully reused 5 times with no appreciable loss of adsorption capacity (<5%) provided it was regenerated with methanol after each cycle. Finally, the adsorption mechanism is based on a hydrophobic interaction effect between the parabens and the cellulose of the materials.

## Supporting Information Available

Effect of initial pH solution on the MPB removal from aqueous solutions by HT-CMC-3 to 30 min; adsorption curves of 4-methylparaben (25 mg/L), 4-propylparaben (5 mg/L) and 4-benzylparaben (1 mg/L); SEM images for (a) HT-CMC-1, (b) HT-CMC-2, (c) HT-CMC-3, (d) HT-CMC-4; TEM images for (a) HT-CMC-1, (b) HT-CMC-2, (c) HT-CMC-3, (d) HT-CMC-4; reuse of HT/CMC-3 for the adsorption of methylparaben; experimental conditions: 50 mL of adsorbate solution, 200 mg of sorbent and 22 °C, and where “0” is fresh material (PDF)

## References

[ref1] Deblonde T.; Cossu-Leguille C.; Hartemann P. Emerging Pollutants in Wastewater: A Review of the Literature. Int. J. Hyg. Environ. Health 2011, 214, 442–448. 10.1016/j.ijheh.2011.08.002. 21885335

[ref2] Barrios-Estrada C.; de Jesús Rostro-Alanis M.; Muñoz-Gutiérrez B. D.; Iqbal H. M. N.; Kannan S.; Parra-Saldívar R. Emergent Contaminants: Endocrine Disruptors and Their Laccase-Assisted Degradation – A Review. Sci. Total Environ. 2018, 612, 1516–1531. 10.1016/j.scitotenv.2017.09.013. 28915546

[ref3] Petric Z.; Ružić J.; Žuntar I. The Controversies of Parabens–an Overview Nowadays. Acta Pharm. 2021, 71, 17–32. 10.2478/acph-2021-0001. 32697748

[ref4] Smarr M. M.; Honda M.; Kannan K.; Chen Z.; Kim S.; Louis G. M. B. Male Urinary Biomarkers of Antimicrobial Exposure and Bi-Directional Associations with Semen Quality Parameters. Reprod. Toxicol. 2018, 77, 103–108. 10.1016/j.reprotox.2018.02.008. 29474822 PMC5878147

[ref5] Darbre P. D.; Harvey P. W. Paraben Esters: Review of Recent Studies of Endocrine Toxicity, Absorption, Esterase and Human Exposure, and Discussion of Potential Human Health Risks. J. Appl. Toxicol. 2008, 28, 561–578. 10.1002/jat.1358. 18484575

[ref6] Pugazhendhi D.; Pope G. S.; Darbre P. D. Oestrogenic Activity of P-hydroxybenzoic Acid (Common Metabolite of Paraben Esters) and Methylparaben in Human Breast Cancer Cell Lines. J. Appl. Toxicol. 2005, 25, 301–309. 10.1002/jat.1066. 16021681

[ref7] Harvey P. W.; Darbre P. Endocrine Disrupters and Human Health: Could Oestrogenic Chemicals in Body Care Cosmetics Adversely Affect Breast Cancer Incidence in Women? A Review of Evidence and Call for Further Research. J. Appl. Toxicol. 2004, 24, 167–176. 10.1002/jat.978. 15211609

[ref8] Mao Q.; Li Q.; Li H.; Yuan S.; Zhang J. Oxidative Paraben Removal with Chlorine Dioxide: Reaction Kinetics and Mechanism. Sep. Purif. Technol. 2020, 237, 116327 10.1016/j.seppur.2019.116327.

[ref9] Song C.; Hu H.; Ao H.; Wu Y.; Wu C. Removal of Parabens and Their Chlorinated By-Products by Periphyton: Influence of Light and Temperature. Environ. Sci. Pollut. Res. 2017, 24, 5566–5575. 10.1007/s11356-016-8301-x. 28032288

[ref10] Pierens X.; Nguyen V. D.; Lauzier T.; Benhabib K. Chemical Actinometry and Paraben Decomposition in Aqueous Solution Utilizing Ultraviolet Radiation Combined with Hydrogen Peroxide. Chem. Pap. 2020, 74, 4283–4294. 10.1007/s11696-020-01237-8.

[ref11] Ding C.; He J.; Xu M.; Wang C. Fabrication of β-Cyclodextrin Modified Mesostructured Silica Coated Multi-Walled Carbon Nanotubes Composites and Application for Paraben Removal. Water Sci. Technol. 2018, 78, 1001–1009. 10.2166/wst.2018.257. 30339525

[ref12] Laghaei M.; Sadeghi M.; Ghalei B.; Dinari M. The Effect of Various Types of Post-Synthetic Modifications on the Structure and Properties of MCM-41 Mesoporous Silica. Prog. Org. Coat. 2016, 90, 163–170. 10.1016/j.porgcoat.2015.10.007.

[ref13] Bin T.; McCrosky L.; Kulshreshtha A. K.; Hem S. L. Adsorption of Esters of P-Hydroxybenzoic Acid by Filter Membranes: Mechanism and Effect of Formulation and Processing Parameters. Pharm. Dev. Technol. 2000, 5, 95–104. 10.1081/PDT-100100524. 10669923

[ref14] Thiebault T. Raw and Modified Clays and Clay Minerals for the Removal of Pharmaceutical Products from Aqueous Solutions: State of the Art and Future Perspectives. Crit. Rev. Environ. Sci. Technol. 2020, 50, 1451–1514. 10.1080/10643389.2019.1663065.

[ref15] Han H.; Rafiq M. K.; Zhou T.; Xu R.; Mašek O.; Li X. A Critical Review of Clay-Based Composites with Enhanced Adsorption Performance for Metal and Organic Pollutants. J. Hazard. Mater. 2019, 369, 780–796. 10.1016/j.jhazmat.2019.02.003. 30851518

[ref16] Martín J.; Orta M.; del M.; Medina-Carrasco S.; Santos J. L.; Aparicio I.; Alonso E. Removal of Priority and Emerging Pollutants from Aqueous Media by Adsorption onto Synthetic Organo-Funtionalized High-Charge Swelling Micas. Environ. Res. 2018, 164, 488–494. 10.1016/j.envres.2018.03.037. 29602092

[ref17] De Oliveira T.; Boussafir M.; Fougère L.; Destandau E.; Sugahara Y.; Guégan R. Use of a Clay Mineral and Its Nonionic and Cationic Organoclay Derivatives for the Removal of Pharmaceuticals from Rural Wastewater Effluents. Chemosphere 2020, 259, 127480 10.1016/j.chemosphere.2020.127480. 32634722

[ref18] Nalawade P.; Aware B.; Kadam V. J.; Hirlekar R. S. Layered Double Hydroxides: A Review. J. Sci. Ind. Res. 2009, 68, 267–272.

[ref19] Wang Q.; O′hare D. Recent Advances in the Synthesis and Application of Layered Double Hydroxide (LDH) Nanosheets. Chem. Rev. 2012, 112, 4124–4155. 10.1021/cr200434v. 22452296

[ref20] Yu J.; Wang Q.; O’Hare D.; Sun L. Preparation of Two Dimensional Layered Double Hydroxide Nanosheets and Their Applications. Chem. Soc. Rev. 2017, 46, 5950–5974. 10.1039/C7CS00318H. 28766671

[ref21] Miyata S. Physico-Chemical Properties of Synthetic Hydrotalcites in Relation to Composition. Clays Clay Miner. 1980, 28, 50–56. 10.1346/CCMN.1980.0280107.

[ref22] Dou Y.; Xu S.; Liu X.; Han J.; Yan H.; Wei M.; Evans D. G.; Duan X. Transparent, Flexible Films Based on Layered Double Hydroxide/Cellulose Acetate with Excellent Oxygen Barrier Property. Adv. Funct. Mater. 2014, 24, 514–521. 10.1002/adfm.201301775.

[ref23] Kang H.; Shu Y.; Li Z.; Guan B.; Peng S.; Huang Y.; Liu R. An Effect of Alginate on the Stability of LDH Nanosheets in Aqueous Solution and Preparation of Alginate/LDH Nanocomposites. Carbohydr. Polym. 2014, 100, 158–165. 10.1016/j.carbpol.2013.07.051. 24188850

[ref24] Dinari M.; Tabatabaeian R. Ultra-Fast and Highly Efficient Removal of Cadmium Ions by Magnetic Layered Double Hydroxide/Guargum Bionanocomposites. Carbohydr. Polym. 2018, 192, 317–326. 10.1016/j.carbpol.2018.03.048. 29691027

[ref25] Dinari M.; Haghighi A.; Asadi P. Facile Synthesis of ZnAl-EDTA Layered Double Hydroxide/Poly(Vinyl Alcohol) Nanocomposites as an Efficient Adsorbent of Cd(II) Ions from the Aqueous Solution. Appl. Clay Sci. 2019, 170, 21–28. 10.1016/j.clay.2019.01.007.

[ref26] Rahmanian O.; Dinari M.; Abdolmaleki M. K. Carbon Quantum Dots/Layered Double Hydroxide Hybrid for Fast and Efficient Decontamination of Cd(II): The Adsorption Kinetics and Isotherms. Appl. Surf. Sci. 2018, 428, 272–279. 10.1016/j.apsusc.2017.09.152.

[ref27] Dinari M.; Shirani M. A.; Maleki M. H.; Tabatabaeian R. Green Cross-Linked Bionanocomposite of Magnetic Layered Double Hydroxide/Guar Gum Polymer as an Efficient Adsorbent of Cr(VI) from Aqueous Solution. Carbohydr. Polym. 2020, 236, 116070 10.1016/j.carbpol.2020.116070. 32172886

[ref28] Dinari M.; Neamati S. Surface Modified Layered Double Hydroxide/Polyaniline Nanocomposites: Synthesis, Characterization and Pb2+ Removal. Colloids Surf., A 2020, 589, 124438 10.1016/j.colsurfa.2020.124438.

[ref29] Rahmanian O.; Dinari M.; Neamati S. Synthesis and Characterization of Citrate Intercalated Layered Double Hydroxide as a Green Adsorbent for Ni^2+^ and Pb^2+^ Removal. Environ. Sci. Pollut. Res. 2018, 25, 36267–36277. 10.1007/s11356-018-3584-8. 30368699

[ref30] Rahmanian O.; Amini S.; Dinari M. Preparation of Zinc/Iron Layered Double Hydroxide Intercalated by Citrate Anion for Capturing Lead (II) from Aqueous Solution. J. Mol. Liq. 2018, 256, 9–15. 10.1016/j.molliq.2018.02.018.

[ref31] Manouchehri M.; Seidi S.; Rouhollahi A.; Noormohammadi H.; Shanehsaz M. Micro Solid Phase Extraction of Parabens from Breast Milk Samples Using Mg-Al Layered Double Hydroxide Functionalized Partially Reduced Graphene Oxide Nanocomposite. Food Chem. 2020, 314, 126223 10.1016/j.foodchem.2020.126223. 31982859

[ref32] Grover A.; Mohiuddin I.; Malik A. K.; Aulakh J. S.; Kukkar D.; Kim K. H. Chitosan-Ni/Fe Layered Double Hydroxide Composites as an Efficient Solid Phase Extraction Sorbent for HPLC-PDA Monitoring of Parabens in Personal Care Products. Chemosphere 2021, 264, 128429 10.1016/j.chemosphere.2020.128429. 33011479

[ref33] Yadollahi M.; Namazi H. Synthesis and Characterization of Carboxymethyl Cellulose/Layered Double Hydroxide Nanocomposites. J. Nanopart. Res. 2013, 15, 1563 10.1007/s11051-013-1563-z.

[ref34] Barkhordari S.; Yadollahi M.; Namazi H. PH Sensitive Nanocomposite Hydrogel Beads Based on Carboxymethyl Cellulose/Layered Double Hydroxide as Drug Delivery Systems. J. Polym. Res. 2014, 21, 454 10.1007/s10965-014-0454-z.

[ref35] Barkhordari S.; Yadollahi M. Carboxymethyl Cellulose Capsulated Layered Double Hydroxides/Drug Nanohybrids for Cephalexin Oral Delivery. Appl. Clay Sci. 2016, 121–122, 77–85. 10.1016/j.clay.2015.12.026.

[ref36] Schwuger M. J.; Lange H. Reciprocal Action between Sodium Carboxymethyl Cellulose and Surfactants. Tenside, Surfactants, Deterg. 1968, 5, 257–259. 10.1515/tsd-1968-059-1001.

[ref37] Miyawaki G.; Patel N. K.; Kostenbaueder H. B. Interaction of preservatives with macromolecules III. Parahydroxybenzoic acid esters in the presence of some hydrophilic polymers. J. Am. Pharm. Assoc. 1959, 48, 315–318. 10.1002/jps.3030480605. 13654083

[ref38] Aramendía M. A.; Avilés Y.; Borau V.; Marinas M.; Ruiz R.; Urbano F. J. Comparative Study of Mg/Al and Mg/Ga Layered Double Hydroxides. Microporous Mesoporous Mater. 1999, 29, 319–328. 10.1016/S1387-1811(98)00345-X.

[ref39] Aramendía M. A.; Borau V.; Jiménez C.; Marinas J. M.; Ruiz J. R.; Urbano F. J. Comparative Study of Mg/M(III) (M=Al, Ga, In) Layered Double Hydroxides Obtained by Coprecipitation and the Sol-Gel Method. J. Solid State Chem. 2002, 168, 156–161. 10.1006/jssc.2002.9655.

[ref40] Mora M.; López M. I.; Jiménez-Sanchidrián C.; Ruiz J. R. Near- and Mid-Infrared Spectroscopy Study of Synthetic Hydrocalumites. Solid State Sci. 2011, 13, 101–105. 10.1016/j.solidstatesciences.2010.10.017.

[ref41] Benito P.; Herrero M.; Labajos F. M.; Rives V. Effect of Post-Synthesis Microwave – Hydrothermal Treatment on the Properties of Layered Double Hydroxides and Related Materials. Appl. Clay Sci. 2010, 48, 218–227. 10.1016/j.clay.2009.11.051.

[ref42] JCPDS. Joint Committee on Powder Diffraction Standards (JCPDS), 1991.

[ref43] Cosano D.; Esquivel D.; Romero F. J.; Jiménez-Sanchidrián C.; Ruiz J. R. Microwave-Assisted Synthesis of Hybrid Organo-Layered Double Hydroxides Containing Cholate and Deoxycholate. Mater. Chem. Phys. 2019, 225, 28–33. 10.1016/j.matchemphys.2018.12.060.

[ref44] Elmoubarki R.; Mahjoubi F. Z.; Elhalil A.; Tounsadi H.; Abdennouri M.; Sadiq M.; Qourzal S.; Zouhri A.; Barka N. Ni/Fe and Mg/Fe Layered Double Hydroxides and Their Calcined Derivatives: Preparation, Characterization and Application on Textile Dyes Removal. J. Mater. Res. Technol. 2017, 6, 271–283. 10.1016/j.jmrt.2016.09.007.

[ref45] Magri V. R.; Duarte A.; Perotti G. F.; Constantino V. R. L. Investigation of Thermal Behavior of Layered Double Hydroxides Intercalated with Carboxymethylcellulose Aiming Bio-Carbon Based Nanocomposites. ChemEngineering 2019, 3, 55 10.3390/chemengineering3020055.

[ref46] de Matos C. S.; Ghimbeu C. M.; Brendlé J.; Limousy L.; Constantino V. R. L. Thermal Decomposition of a Layered Double Hydroxide as a Bottom up Approach for the Synthesis of Metallic Nanoparticles Embedded in Carbon Structures. New J. Chem. 2020, 44, 16721–16732. 10.1039/D0NJ01938K.

[ref47] Park D. H.; Hwang S. J.; Oh J. M.; Yang J. H.; Choy J. H. Polymer-Inorganic Supramolecular Nanohybrids for Red, White, Green, and Blue Applications. Prog. Polym. Sci. 2013, 38, 1442–1486. 10.1016/j.progpolymsci.2013.05.007.

[ref48] Cosano D.; Esquinas C.; Jiménez-Sanchidrián C.; Ruiz J. R. Use of Raman Spectroscopy to Assess the Efficiency of MgAl Mixed Oxides in Removing Cyanide from Aqueous Solutions. Appl. Surf. Sci. 2016, 364, 428–433. 10.1016/j.apsusc.2015.12.181.

[ref49] Mora M.; Jiménez-Sanchidrián C.; Ruiz J. R. Raman Spectroscopy Study of Layered-Double Hydroxides Containing Magnesium and Trivalent Metals. Mater. Lett. 2014, 120, 193–195. 10.1016/j.matlet.2014.01.085.

[ref50] Cosano D.; Jiménez-Sanchidrián C.; Ruiz J. R. Vibrational Spectroscopic Study of Sol–Gel Layered Double Hydroxides Containing Different Tri- and Tetravalent Cations. J. Sol-Gel Sci. Technol. 2015, 76, 614–620. 10.1007/s10971-015-3812-3.

[ref51] Cosano D.; Esquivel D.; Romero-Salguero F. J.; Jiménez-Sanchidrián C.; Ruiz J. R. Oleate Epoxidation in a Confined Matrix of Hydrotalcite. ACS Omega 2020, 5, 619–625. 10.1021/acsomega.9b03212. 31956810 PMC6964289

[ref52] Mul G.; Moulijn J. A.; Pe J. In Situ Fourier Transform Infrared and Laser Raman Spectroscopic Study of the Thermal Decomposition of Co-Al and Ni-Al Hydrotalcites. Vib. Spectrosc. 2001, 27, 75–88. 10.1016/S0924-2031(01)00119-9.

[ref53] Kloprogge J. T.; Hickey L.; Frost R. Synthesis and Spectroscopic Characterization of Deuterated Hydrotalcite. J. Mater. Sci. Lett. 2002, 21, 603–605. 10.1023/A:1015655018529.

[ref54] Rosca C.; Popa M. I.; Lisa G.; Chitanu G. C. Interaction of Chitosan with Natural or Synthetic Anionic Polyelectrolytes. 1. The Chitosan-Carboxymethylcellulose Complex. Carbohydr. Polym. 2005, 62, 35–41. 10.1016/j.carbpol.2005.07.004.

[ref55] Olszówka J. E.; Karcz R.; Bielańska E.; Kryściak-Czerwenka J.; Napruszewska B. D.; Sulikowski B.; Socha R. P.; Gaweł A.; Bahranowski K.; Olejniczak Z.; Serwicka E. M. New Insight into the Preferred Valency of Interlayer Anions in Hydrotalcite-like Compounds: The Effect of Mg/Al Ratio. Appl. Clay Sci. 2018, 155, 84–94. 10.1016/j.clay.2018.01.013.

[ref56] Yadollahi M.; Namazi H. Synthesis and Characterization of Carboxymethyl Cellulose/Layered Double Hydroxide Nanocomposites. J. Nanopart. Res. 2013, 15, 1563 10.1007/s11051-013-1563-z.

[ref57] Ghimbeu C. M.; Zhang B.; de Yuso A. M.; Réty B.; Tarascon J. M. Valorizing Low Cost and Renewable Lignin as Hard Carbon for Na-Ion Batteries: Impact of Lignin Grade. Carbon 2019, 153, 634–647. 10.1016/j.carbon.2019.07.026.

[ref58] Hameed B. H. Equilibrium and Kinetics Studies of 2,4,6-Trichlorophenol Adsorption onto Activated Clay. Colloids Surf., A 2007, 307, 45–52. 10.1016/j.colsurfa.2007.05.002.

[ref59] Yuan Q.; Chi Y.; Yu N.; Zhao Y.; Yan W.; Li X.; Dong B. Amino-Functionalized Magnetic Mesoporous Microspheres with Good Adsorption Properties. Mater. Res. Bull. 2014, 49, 279–284. 10.1016/j.materresbull.2013.08.063.

[ref60] Correa F. G.; Gómez J. S. Kinetic and Thermodynamic Parameters of 99Mo Sorption on Thermally Treated Hydrotalcite. J. Radioanal. Nucl. Chem. 2006, 268, 95–101. 10.1007/s10967-006-0130-9.

[ref61] Saghir S.; Xiao Z. Hierarchical Mesoporous ZIF-67@LDH for Efficient Adsorption of Aqueous Methyl Orange and Alizarine Red S. Powder Technol. 2021, 377, 453–463. 10.1016/j.powtec.2020.09.006.

[ref62] Özcan A. S.; Erdem B.; Özcan A. Adsorption of Acid Blue 193 from Aqueous Solutions onto Na-Bentonite and DTMA-Bentonite. J. Colloid Interface Sci. 2004, 280, 44–54. 10.1016/j.jcis.2004.07.035. 15476772

[ref63] Ivančev-Tumbas I.; Lužanin Z.; Česen M.; Bogunović M.; Sekulić T. D.; Heath D.; Heath E. Insight into Selected Emerging Micropollutant Interactions with Wastewater Colloidal Organic Carbon: Implications for Water Treatment and Analysis. Environ. Sci. Pollut. Res. 2021, 28, 59368–59381. 10.1007/s11356-020-11309-7. 33146819

[ref64] Rostvall A.; Zhang W.; Dürig W.; Renman G.; Wiberg K.; Ahrens L.; Gago-Ferrero P. Removal of Pharmaceuticals, Perfluoroalkyl Substances and Other Micropollutants from Wastewater Using Lignite, Xylit, Sand, Granular Activated Carbon (GAC) and GAC+Polonite in Column Tests – Role of Physicochemical Properties. Water Res. 2018, 137, 97–106. 10.1016/j.watres.2018.03.008. 29544207

[ref65] Blaug S. M.; Ashan S. S. Interaction of Parabens with Nonionic Macromolecules. J. Pharm. Sci. 1961, 50, 441–443. 10.1002/jps.2600500516.

[ref66] Zhu F. D.; Choo K. H.; Chang H. S.; Lee B. Interaction of Bisphenol A with Dissolved Organic Matter in Extractive and Adsorptive Removal Processes. Chemosphere 2012, 87, 857–864. 10.1016/j.chemosphere.2012.01.026. 22330311

[ref67] Kurup T. R. R.; Wan L. S. C.; Chan L. W. Interaction of Preservatives with Macromolecules. Part II. Cellulose Derivatives. Pharm. Acta Helv. 1995, 70, 187–193. 10.1016/0031-6865(95)00020-A. 1470635

