# Supplementary material for: Carboxymethylcellulose/Hydrotalcite Bionanocomposites as Paraben Sorbents
Source: Langmuir. 2023 Apr 6;39(15):5294–305. doi: 10.1021/acs.langmuir.2c03265 (PMC10849270; doi:10.1021/acs.langmuir.2c03265)

Supporting Information

Carboxymethylcellulose/hydrotalcite bionanocomposites as paraben sorbents

Daniel Cosano*, Dolores Esquivel, Francisco J. Romero-Salguero, César Jiménez-Sanchidrián and José Rafael Ruiz*

Departamento de Química Orgánica, Instituto Químico para la Energía y el Medioambiente (IQUEMA), Facultad de Ciencias, Universidad de Córdoba, Campus de Rabanales, Edificio Marie Curie, E-14071 Córdoba, Spain

*Corresponding authors. D. Cosano: q92cohid@uco.es; J. R. Ruiz: qo1ruarj@uco.es

Figure S1. Effect of initial pH solution on the MPB removal from aqueous solutions by HT-CMC-3 to 30 min.

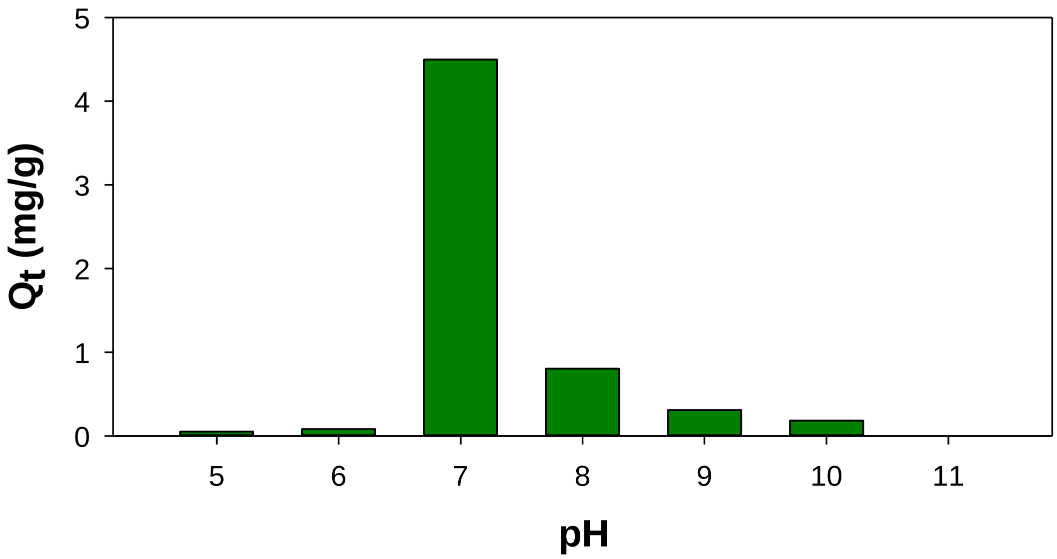

Figure S2. Adsorption curves of 4-methylparaben (25 mg/L), 4-propylparaben (5 mg/L) and 4-benzylparaben (1 mg/L).

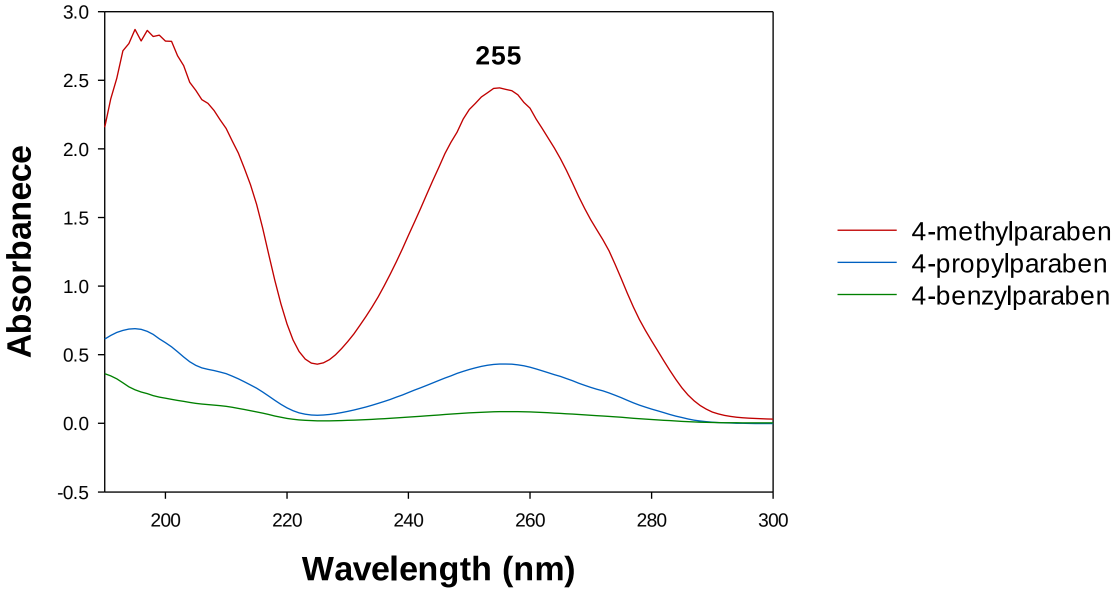

Figure S3. SEM images for (a) HT-CMC-1, (b) HT-CMC-2, (c) HT-CMC-3, (d) HT-CMC-4

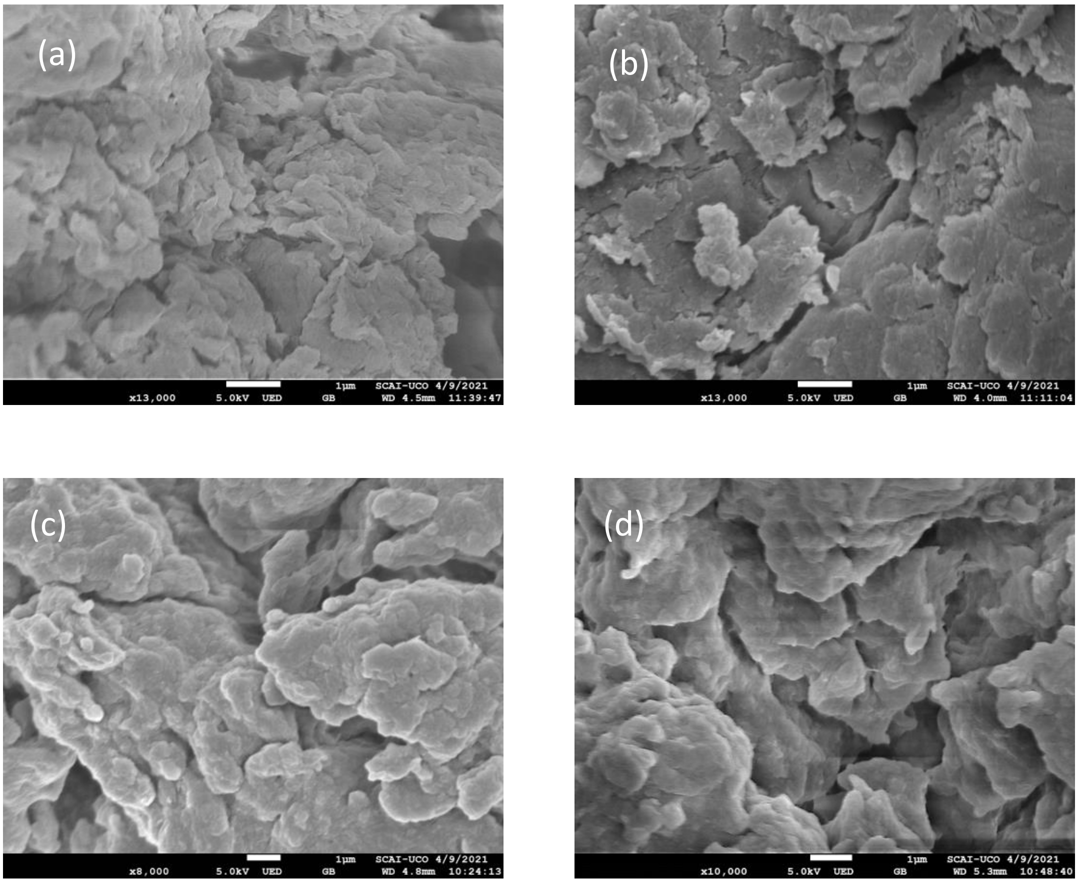

Figure S4. TEM images for (a) HT-CMC-1, (b) HT-CMC-2, (c) HT-CMC-3, (d) HT-CMC-4

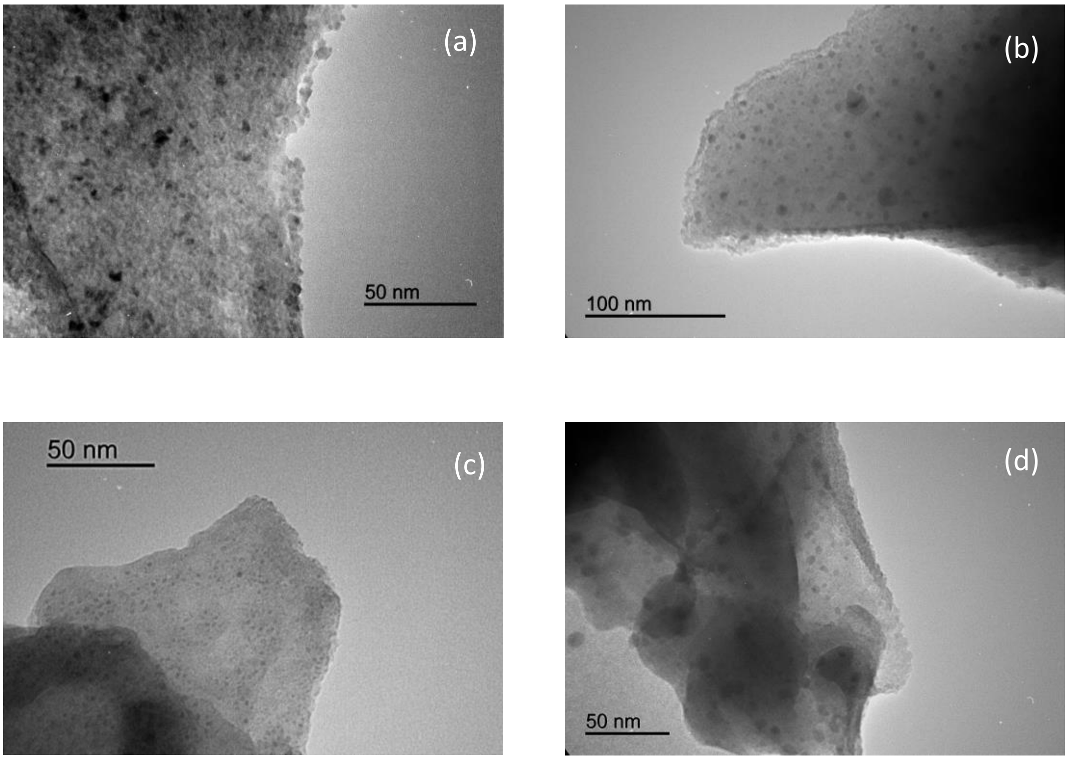

Figure S5. Reuse of HT/CMC-3 for the adsorption of methylparaben. Experimental conditions: 50 mL of adsorbate solution, 200 mg of sorbent and 22 °C. Where "0" is fresh material.

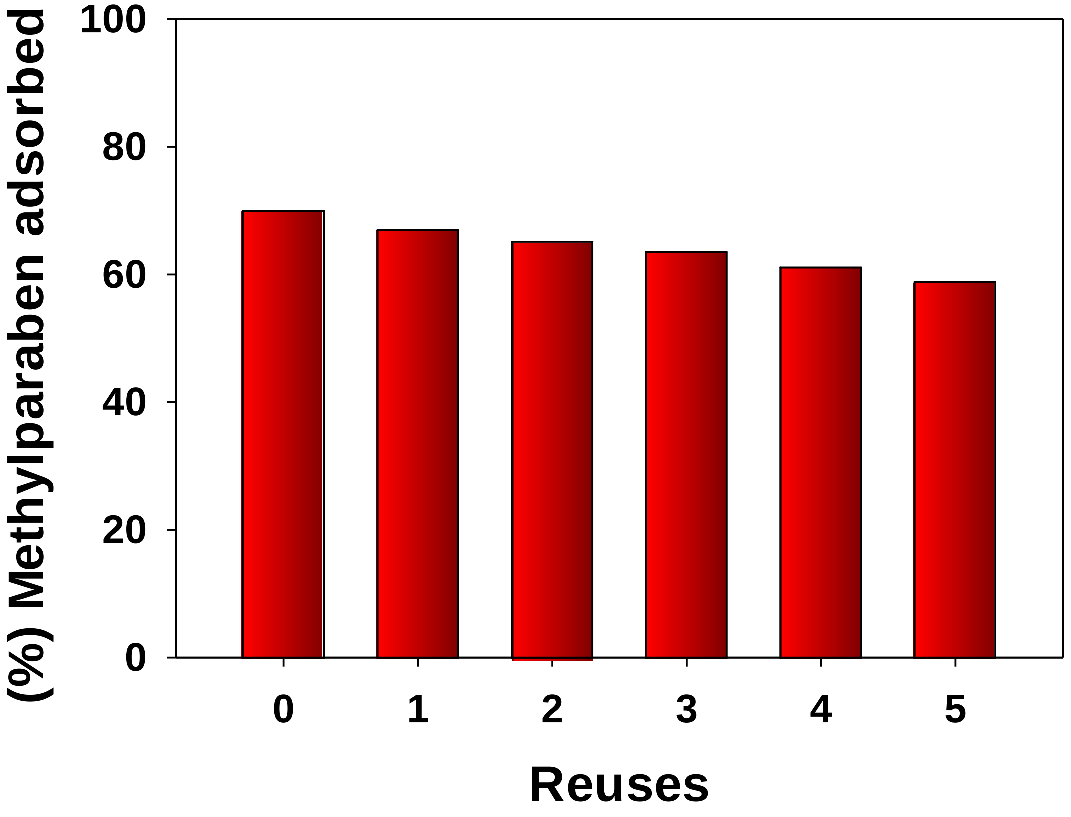

Supplement: Supplementary file 1 — la2c03265_si_001.pdf [file la2c03265_si_001.pdf]
